# Comparing Temporal Trends in Aesthetic Surgery Fellowship Match Statistics in Plastic Surgery, Facial Plastic Surgery, and Oculofacial Surgery

**DOI:** 10.1093/asjof/ojaf123

**Published:** 2025-10-04

**Authors:** Isaac B James, Pierce L Janssen, Elad Fraiman, Marjorie C Kragel, Huijun Xiao, James E Zins

## Abstract

**Background:**

Aesthetic plastic surgery fellowships endorsed by The Aesthetic Society participated in the San Francisco (SF) Match in 2017 to 2018. Despite streamlined applications and increases in available positions, participating applicants did not increase through the 2022 match cycle.

**Objectives:**

The authors of this study compare match statistics for aesthetic plastic surgery, facial plastic surgery, and oculofacial surgery, with analysis extended through the 2025 match cycle.

**Methods:**

SF Match data for aesthetic plastic surgery (2018-2025), facial plastic surgery (2018-2024), and oculofacial surgery (2018-2024) were analyzed. Relevant data included the number of participating programs, positions, applicants, filled positions, and match rates. The 2017 to 2024 Accreditation Council for Graduate Medical Education Data Resource Books provided resident graduation data by subspecialty.

**Results:**

Aesthetic plastic surgery positions increased significantly (*P* = .047) between 2018 and 2022, whereas applicant numbers remained unchanged (*P* > .99). Participating aesthetic plastic surgery applicants more than doubled from 28 in 2022 to 59 in 2025 (*P* = .026), whereas available position numbers slightly decreased (*P* > .99). No significant changes occurred in filled positions (ie, applicants matched), fill rates, or match rates between match cycles. Facial plastic surgery and oculofacial surgery fellowship trends were stable, with no significant changes in these metrics.

**Conclusions:**

Efforts to improve the postgraduate plastic surgery aesthetic application process and training model have had a significant, although delayed, impact on application numbers. Applications to facial plastic surgery and oculofacial surgery fellowships have seen only nominal increases during the same time period. Previously, facial plastic surgery fellowships greatly outnumbered aesthetic plastic surgery fellowships. This gap has now significantly narrowed. Oculofacial surgery fellowships remain highly competitive and limited in numbers.

**Level of Evidence:**

5 (Therapeutic) 
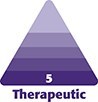

Aesthetic plastic surgery fellowships serve as a major pathway for additional training following plastic surgery residency. Residents may seek this additional experience for a number of reasons. Some may feel that the fellowship will enhance their opportunity to join a group practice. Others may find it valuable because they plan to practice in a competitive area or wish to refine their skills in areas of perceived training weakness.^1-3^ Other surgical specialties have also established postresidency fellowships to meet growing demand for aesthetic surgery. Specifically, further aesthetic training opportunities exist in facial plastic surgery fellowships after otolaryngology (Oto-HNS) residency and oculofacial (formerly oculoplastic) surgery after ophthalmology residency.^4,5^

The Endorsed Aesthetic Fellowship sponsored by The Aesthetic Society joined the San Francisco (SF) match beginning in the 2017 to 2018 application cycle. This was done to streamline the fellowship application process and make it more user-friendly. At about the same time, The Aesthetic Society significantly increased the number of endorsed fellowship programs and initiated additional steps to enhance program quality. In a previous study, our group investigated the early effects of these changes on the aesthetic plastic surgery fellowship application process. Specifically, we analyzed the changes in program numbers, fellowship positions, fellowship applications, and fill rates from 2018 (the first match year) through 2022. We expected to see an increase in the number of fellowship applications and match rates given the increase in program numbers and positions as well as the other positive steps taken by The Aesthetic Society. However, this proved not to be the case.^4^ Although the number of programs and program positions increased, the number of aesthetic plastic surgery fellowship applicants did not change significantly. At the time of publication, we suggested that this finding was because of the finite number of potential applications. The application pool is limited, with prospective fellows being selected almost exclusively from American Board of Plastic Surgery (ABPS) approved residencies. For example, as outlined in the previous publication, 221 plastic surgery residents graduated in 2022. Of these graduates, 106 (48.0%) pursued fellowship training in one of the plastic surgery subspecialties. If 1 was to assume these graduates chose equally among the 4 major subspecialties (hand, microsurgery, craniofacial, or aesthetic surgery), this would yield ∼27 applications per subspecialty. This was very close the number of aesthetic fellow applications seen during the 2018 to 2022 match period. We therefore concluded that aesthetic fellowship applications would only theoretically increase if those choosing not to pursue fellowships were convinced to do so, or if residents choosing to pursue other plastic surgery fellowships were convinced to choose aesthetic surgery instead. We therefore predicted that aesthetic fellowship applications would not increase appreciably over time.

Public SF Match data are now available through the 2024 match cycle for facial plastic surgery and oculofacial surgery fellowships, and through the 2025 match cycle for aesthetic plastic surgery fellowships. Accreditation Council for Graduate Medical Education (ACGME) Data Resource Book data are now available through the 2023 to 2024 graduation year.^6-12^ The purpose of this report is (1) to further analyze the trends in the aesthetic surgery fellowship application process given that additional years of data are now available and (2) to compare these results to the trends in program numbers, positions, applications, and match rates of the endorsed fellowships to similar numbers in facial plastic and oculofacial surgery fellowships.

## METHODS

This study was conducted as a cross-sectional retrospective review of prospectively collected data and was approved by the Cleveland Clinic Institutional Review Board (IRB number 22-365). Funding was provided as an Aesthetic Foundation Interim Research Grant through the Aesthetic Foundation under grant number 2021-Q2C.

### Data Collection

SF Match data for all United States postgraduate Aesthetic Society–endorsed aesthetic surgery fellowships were obtained from 2018 to 2024. Additional data for aesthetic plastic surgery fellowships, but not facial plastic surgery or oculofacial surgery, was available for the 2025 match year. Comparison data for postgraduate facial plastic surgery and oculofacial surgery fellowships were also obtained from SF Match data over the relevant time periods. Program inclusion was based on participation in the SF Match. Plastic surgery aesthetic fellowships not endorsed by The Aesthetic Society were excluded from the study. Applications and programs outside of the SF Match were excluded, as were incomplete and unsubmitted applications. Data collected included number of applicants, number of fellowship programs, number of fellowship positions, and number of positions filled. Relevant match rates and position fill rates were calculated from this raw data.

Data regarding graduating residents were collected from the annually published ACGME Data Resource Books from 2017 to 2018 through 2023 to 2024.^6-12^

### Statistics

To evaluate temporal trends in counts endpoints, separate univariate Poisson regression models with a log link function were fitted for each specialty or fellowship type. Additional Poisson regression models were utilized to assess trends in fill and match rates, incorporating offsets to account for exposure: the total number of positions available (for filled and unfilled positions) and the total number of participating applicants (for matched and unmatched applicants). Poisson regression models with offset terms were utilized instead of binomial models here because of convergence issues arising from zero counts in some cases. Time was modeled as a categorical variable to allow for year-to-year comparisons. Model results are reported as incidence rate ratios (IRRs) with corresponding 95% CIs. A global *P*-value for time was derived from a Type III Wald test to assess whether the time variable as a whole was significantly associated with the outcome. Pairwise comparisons on interested endpoints between specific years were performed and adjusted for multiple testing by the Tukey method. Of note, modeling unfilled positions—either as an absolute count or rate—was statistically unfeasible, as several years had zero unfilled positions, which leads to complete separation in regression modeling.

To examine the association between the number of graduating residents and match outcomes, univariate Poisson regression models with a log link were utilized to model matched and unmatched counts by fellowship type. Similar Poisson models with an offset for total participating applicants were utilized to assess the association between the number of graduating residents and match rates (matched and unmatched). We specifically utilized Poisson regression models to appropriately evaluate count data with temporal correlation, which was not previously done in our study or in a recently published study by Katz et al.^13^ All results are presented as IRRs with 95% CIs.

All models were assessed through standard diagnostic procedures to verify appropriate model fit and ensure the validity of the result inferences. The overall significance level is .05, which means a 2-sided *α* of <.05 was considered statistically significant for this study. Data were managed and analyzed using R version 4.3.1 (R Foundation for Statistical Computing, Vienna, Austria).

## RESULTS

### Aesthetic Plastic Surgery Fellowships (Plastic Surgery)

**Figure 1.**
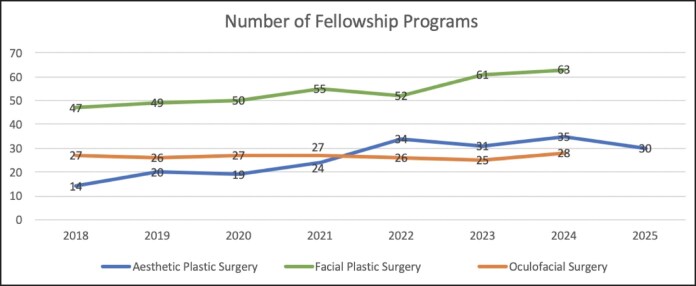
Number of participating fellowship programs by subspecialty.

**Figure 2.**
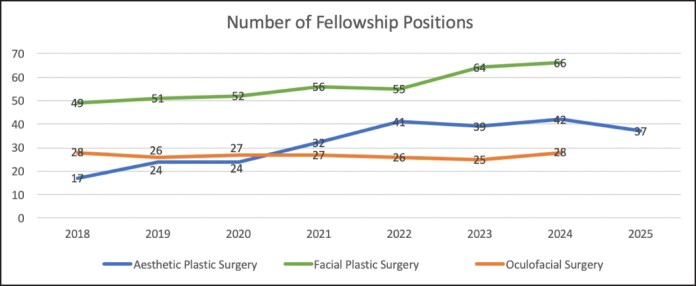
Number of available fellowship positions by subspecialty.

**Figure 3.**
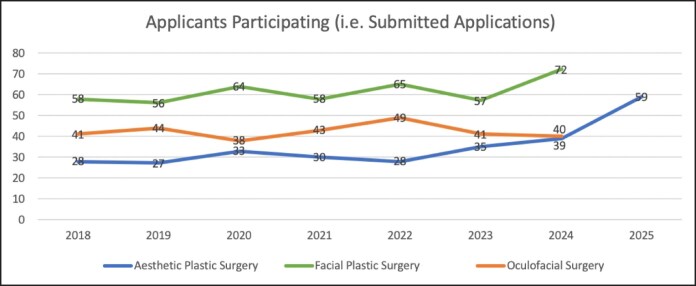
Number of participating fellowship applicants by subspecialty.

Participating aesthetic plastic surgery fellowship programs increased from 14 in 2018 to 34 in 2022 (+142.9%) and then to 30 in 2025 (+114.3% compared with 2018; Table 1; Figure 1). There were no significant differences in the number of participating programs between match cycles (Table 2). Total available positions increased from 17 in 2018 to 41 in 2022 (+141.2%) to 37 in 2025 (+117.6% compared with 2018; Table 1; Figure 2). There was a significant increase in available positions between 2018 and 2022 (*P* = .047), but not between 2018 and 2025 (*P* = .14) or between 2022 and 2025 (*P* > .99; Table 2). Participating aesthetic plastic surgery applicants remained stable at 28 between 2018 and 2022, despite the significant increase in available position during this premoratorium period. Applicants then increased from 28 in 2022 to 59 in 2025 (+110.7%) while available positions numbers slightly decreased (Table 1; Figure 3). There was a significant increase in the number of participating applicants between both the 2018 to 2025 (*P* = .026) and 2022 to 2025 (*P* = .026) time periods, but not between 2018 and 2022 (*P* > .99; Table 2). The number of unmatched aesthetic plastic surgery applicants decreased from 11 in 2018 (39.3% unmatched rate) to 3 in 2022 (10.7% unmatched rate) despite an equal number of participating applicants for each of these cycles, but then increased to 26 in 2025 (44.1% unmatched rate; Table 1). There was a significant increase in the number of unmatched applicants only between 2022 and 2025 (*P* = .009; Table 2). There were no significant differences in the number of positions filled (ie, applicants matched), position fill rate, applicant match rate, or applicant unmatched rate between match cycles (Table 2).

**Table 1.** Aesthetic Plastic Surgery Fellowship Data, San Francisco Match, 2018-2025

Year	2018	2019	2020	2021	2022	2023	2024	2025
Programs participating	14	20	19	24	34	31	35	30
Positions offered	17	24	24	32	41	39	42	37
Positions filled	17	19	20	22	25	26	31	33
Positions unfilled	0	5	4	10	16	13	11	4
Position fill rate	100.0%	79.2%	83.3%	68.8%	61.0%	66.7%	73.8%	89.2%
Position unfilled rate	0.0%	20.8%	16.7%	31.2%	39.0%	33.3%	26.2%	10.8%
Applicants participating	28	27	33	30	28	35	39	59
Applicants matched	17	19	20	22	25	26	31	33
Applicants unmatched	11	8	13	8	3	9	8	26
Applicant match rate	60.7%	70.4%	60.6%	73.3%	89.3%	74.3%	79.5%	55.9%
Applicant unmatched rate	39.3%	29.6%	39.4%	26.7%	10.7%	25.7%	20.5%	44.1%

**Table 2.** Aesthetic Plastic Surgery Statistical Analysis

	Match cycle comparison	IRR	*P*-value
Programs participating	2022 vs 2018	2.43 (0.93, 6.36)	.096
	2025 vs 2018	2.14 (0.80, 5.72)	.26
	2025 vs 2022	0.88 (0.41, 1.89)	>.99
Positions offered	2022 vs 2018	2.41 (1.01, 5.78)	.047
	2025 vs 2018	2.18 (0.90, 5.29)	.14
	2025 vs 2022	0.9 (0.45, 1.79)	>.99
Positions filled	2022 vs 2018	1.47 (0.57, 3.81)	.92
	2025 vs 2018	1.94 (0.79, 4.8)	.34
	2025 vs 2022	1.32 (0.59, 2.95)	.97
Position fill rate	2022 vs 2018	0.61 (0.24, 1.58)	.77
	2025 vs 2018	0.89 (0.36, 2.2)	>.99
	2025 vs 2022	1.46 (0.65, 3.27)	.84
Applicants participating	2022 vs 2018	1.00 (0.44, 2.25)	>.99
	2025 vs 2018	2.11 (1.05, 4.22)	.026
	2025 vs 2022	2.11 (1.05, 4.22)	.026
Applicants matched	2022 vs 2018	1.47 (0.57, 3.81)	.92
	2025 vs 2018	1.94 (0.79, 4.8)	.34
	2025 vs 2022	1.32 (0.59, 2.95)	.97
Applicant match rate	2022 vs 2018	1.47 (0.57, 3.81)	.92
	2025 vs 2018	0.92 (0.37, 2.28)	>.99
	2025 vs 2022	0.63 (0.28, 1.4)	.64
Applicants unmatched	2022 vs 2018	0.27 (0.04, 1.96)	.49
	2025 vs 2018	2.36 (0.79, 7.03)	.25
	2025 vs 2022	8.67 (1.37, 55.01)	.009
Applicant unmatched rate	2022 vs 2018	0.27 (0.04, 1.96)	.49
	2025 vs 2018	1.12 (0.38, 3.34)	>.99
	2025 vs 2022	4.11 (0.65, 26.11)	.28

Univariate Poisson regression model was conducted between the number of position offered and year category for each fellowship type. IRR, incidence rate ratio. Estimated IRR was calculated as the multiplicative change in expected counts comparing the specified year to the reference year (lower 95% CI, upper 95% CI).

### Facial Plastic Surgery (Oto-HNS)

Both participating facial plastic surgery fellowship programs and positions increased only modestly during the study period. Facial plastic surgery programs increased from 47 in 2018 to 52 in 2022 (+10.6%) to 63 in 2024 (+34.0% compared with 2018; Table 3; Figure 1). Total available positions increased from 49 in 2018 to 55 in 2022 (+12.2%) to 66 in 2024 (+34.7% compared with 2018; Table 3; Figure 2). There were no significant differences in the number of programs or positions between match cycles (Table 4). The number of participating facial plastic surgery applicants followed a similar trend, with modest increase from 58 in 2018 to 65 in 2022 (+12.1% compared with 2018) to 72 in 2024 (+24.1% compared with 2018; Table 3; Figure 3). There were no significant differences in the number of participating applicants between match cycles (Table 4). There were no significant differences in the number of positions filled (ie, applicants matched), applicants unmatched, position fill rate, applicant match rate, or applicant unmatched rate between match cycles (Table 4).

**Table 3.** Facial Plastic Surgery Fellowship Data, San Francisco Match, 2018 to 2024

Year	2018	2019	2020	2021	2022	2023	2024	2025
Programs participating	47	49	50	55	52	61	63	N/A
Positions offered	49	51	52	56	55	64	66	N/A
Positions filled	46	46	51	50	55	52	62	N/A
Positions unfilled	3	5	1	6	0	12	4	N/A
Position fill rate	93.9%	90.2%	98.1%	89.3%	100.0%	81.3%	93.9%	N/A
Position unfilled rate	6.1%	9.8%	1.9%	10.7%	0.0%	18.8%	6.1%	N/A
Applicants participating	58	56	64	58	65	57	72	N/A
Applicants matched	46	46	51	50	55	52	62	N/A
Applicants unmatched	12	10	13	8	10	5	10	N/A
Applicant match rate	79.3%	82.1%	79.7%	86.2%	84.6%	91.2%	86.1%	N/A
Applicant unmatched rate	20.7%	17.9%	20.3%	13.8%	15.4%	8.8%	13.9%	N/A

**Table 4.** Facial Plastic Surgery Statistical Analysis

	Match cycle comparison	IRR	*P*-value
Programs participating	2022 vs 2018	1.11 (0.61, 2.00)	>.99
	2024 vs 2018	1.34 (0.76, 2.37)	.73
	2024 vs 2022	1.21 (0.70, 2.10)	.95
Positions offered	2022 vs 2018	1.12 (0.63, 2.00)	>.99
	2024 vs 2018	1.35 (0.77, 2.35)	.70
	2024 vs 2022	1.20 (0.70, 2.06)	.95
Positions filled	2022 vs 2018	1.20 (0.66, 2.15)	.97
	2024 vs 2018	1.35 (0.76, 2.39)	.72
	2024 vs 2022	1.13 (0.65, 1.95)	>.99
Position fill rate	2022 vs 2018	1.07 (0.59, 1.92)	>.99
	2024 vs 2018	1.00 (0.56, 1.78)	>.99
	2024 vs 2022	0.94 (0.54, 1.62)	>.99
Applicants participating	2022 vs 2018	1.12 (0.66, 1.91)	>.99
	2024 vs 2018	1.24 (0.74, 2.09)	.88
	2024 vs 2022	1.11 (0.67, 1.83)	>.99
Applicants matched	2022 vs 2018	1.20 (0.66, 2.15)	.97
	2024 vs 2018	1.35 (0.76, 2.39)	.72
	2024 vs 2022	1.13 (0.65, 1.95)	>.99
Applicant match rate	2022 vs 2018	1.07 (0.59, 1.92)	>.99
	2024 vs 2018	1.09 (0.61, 1.93)	>.99
	2024 vs 2022	1.02 (0.59, 1.76)	>.99
Applicants unmatched	2022 vs 2018	0.83 (0.24, 2.94)	>.99
	2024 vs 2018	0.83 (0.24, 2.94)	>.99
	2024 vs 2022	1.00 (0.27, 3.74)	>.99
Applicant unmatched rate	2022 vs 2018	0.74 (0.21, 2.63)	>.99
	2024 vs 2018	0.67 (0.19, 2.37)	.97
	2024 vs 2022	0.90 (0.24, 3.37)	>.99

Univariate Poisson regression model was conducted between the number of position offered and year category for each fellowship type. IRR, incidence rate ratio. Estimated IRR was calculated as the multiplicative change in expected counts comparing the specified year to the reference year (lower 95% CI, upper 95% CI).

### Oculofacial Surgery (Ophthalmology)

**Figure 4.**
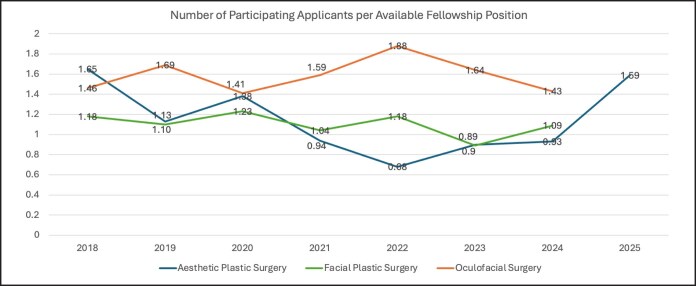
Number of participating applicants per available fellowship position.

Participating oculofacial surgery fellowship programs decreased slightly from 27 in 2018 to 26 in 2022 (−3.7%), then increased to 28 in 2024 (+3.7% compared with 2018; Table 5; Figure 1). Total positions available decreased slightly from 28 in 2018 to 26 in 2022 (−7.1%), then increased back to 28 in 2024 (no change compared with 2018; Table 5; Figure 2). There were no significant differences in the number of programs or positions between match cycles (Table 6). The number of participating oculofacial surgery applicants increased from 41 in 2018 to 49 in 2022 (+19.5%), then decreased to 40 in 2024 (−2.4% compared with 2018; Table 5; Figure 3). There were no significant differences in the number of participating applicants between match cycles (Table 6). There were no significant differences in the number of positions filled (ie, applicants matched), applicants unmatched, position fill rate, applicant match rate, or applicant unmatched rate between match cycles (Table 6). The number of participating applicants per available aesthetic fellowship position (by specialty) is shown in Figure 4.

**Table 5.** Oculofacial Surgery Fellowship Data, San Francisco Match, 2018 to 2024

Year	2018	2019	2020	2021	2022	2023	2024	2025
Programs participating	27	26	27	27	26	25	28	N/A
Positions offered	28	26	27	27	26	25	28	N/A
Positions filled	27	26	24	27	26	25	28	N/A
Positions unfilled	1	0	3	0	0	0	0	N/A
Position fill rate	96.4%	100.0%	88.9%	100.0%	100.0%	100.0%	100.0%	N/A
Position unfilled rate	3.6%	0.0%	11.1%	0.0%	0.0%	0.0%	0.0%	N/A
Applicants participating	41	44	38	43	49	41	40	N/A
Applicants matched	27	26	24	27	26	25	28	N/A
Applicants unmatched	14	18	14	16	23	16	17	N/A
Applicant match rate	65.9%	59.1%	63.2%	62.8%	53.1%	61.0%	70.0%	N/A
Applicant unmatched rate	34.1%	40.9%	36.8%	37.2%	46.9%	39.0%	42.5%	N/A

N/A, not applicable.

**Table 6.** Oculofacial Surgery Statistical Analysis

	Match cycle comparison	IRR	*P*-value
Programs participating	2022 vs 2018	0.96 (0.43, 2.16)	>.99
	2024 vs 2018	1.04 (0.47, 2.30)	>.99
	2024 vs 2022	1.08 (0.48, 2.40)	>.99
Positions offered	2022 vs 2018	0.93 (0.42, 2.07)	>.99
	2024 vs 2018	1.00 (0.45, 2.20)	>.99
	2024 vs 2022	1.08 (0.48, 2.40)	>.99
Positions filled	2022 vs 2018	0.96 (0.43, 2.16)	>.99
	2024 vs 2018	1.04 (0.47, 2.30)	>.99
	2024 vs 2022	1.08 (0.48, 2.40)	>.99
Position fill rate	2022 vs 2018	1.04 (0.46, 2.33)	>.99
	2024 vs 2018	1.04 (0.47, 2.30)	>.99
	2024 vs 2022	1.00 (0.45, 2.23)	>.99
Applicants participating	2022 vs 2018	1.2 (0.64, 2.23)	.98
	2024 vs 2018	0.98 (0.51, 1.88)	>.99
	2024 vs 2022	0.82 (0.44, 1.53)	.96
Applicants matched	2022 vs 2018	0.96 (0.43, 2.16)	>.99
	2024 vs 2018	1.04 (0.47, 2.30)	>.99
	2024 vs 2022	1.08 (0.48, 2.40)	>.99
Applicant match rate	2022 vs 2018	0.81 (0.36, 1.81)	.99
	2024 vs 2018	1.06 (0.48, 2.35)	>.99
	2024 vs 2022	1.32 (0.59, 2.94)	.95
Applicants unmatched	2022 vs 2018	1.64 (0.60, 4.46)	.77
	2024 vs 2018	1.21 (0.42, 3.52)	>.99
	2024 vs 2022	0.74 (0.29, 1.90)	.97
Applicant unmatched rate	2022 vs 2018	1.37 (0.51, 3.73)	.97
	2024 vs 2018	1.24 (0.43, 3.61)	>.99
	2024 vs 2022	0.91 (0.35, 2.32)	>.99

Univariate Poisson regression model was conducted between the number of position offered and year category for each fellowship type. IRR, incidence rate ratio. Estimated IRR was calculated as the multiplicative change in expected counts comparing the specified year to the reference year (lower 95% CI, upper 95% CI).

### Association With Graduating Residents

**Figure 5.**
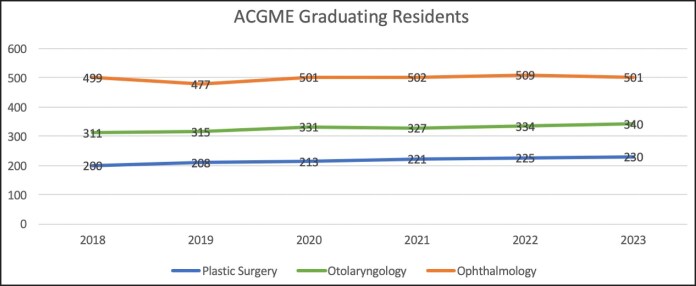
Number of graduating residents by subspecialty. ACGME, Accreditation Council for Graduate Medical Education.

The number of graduating plastic surgery residents increased from 200 to 230 (+15.0%) from 2018 to 2023. Otolaryngology graduates increased from 311 to 334 (+9.3%), and ophthalmology residency graduates increased from 499 to 501 (+0.4%) over the same period (Figure 5).^12^ These changes were not statistically significant for any of the 3 specialties, as demonstrated by *P*-values >.70 and IRRs ∼1.00 (Supplemental Tables 1-3). Of note, 2024 graduation data are not yet available at the time of writing. Poisson regression models were utilized to evaluate whether the number of graduating residents was associated with the number match rate and unmatched rate for each fellowship type. Across all 3 fellowship types, the number of graduating residents was not significantly associated with match rate, as demonstrated by *P*-values >.29 and IRRs ∼1.00, or unmatched rate, as demonstrated by *P*-values >.097 and IRRs ∼1.00. These findings suggest that match rates and unmatched rates were relatively stable, even as the graduating cohorts varied in size over time.

## DISCUSSION

Several studies have analyzed the desirability of postgraduate aesthetic surgery training over time.^4,13^ A recent publication by our group studied aesthetic plastic surgery fellowship program numbers, positions, and match rates from 2018 to 2022, and compared these findings with similar data for the other major plastic surgery fellowships (ie, craniofacial surgery, microsurgery, hand surgery).^4^ This was done in an attempt to evaluate the effect of the recently initiated SF Match and other steps taken by The Aesthetic Society to improve the quality of the fellowship experience. Although the number of aesthetic plastic surgery fellowship programs and positions increased significantly from 2018 to 2022 the number of applications did not. This resulted in a surplus of available positions relative to participating applicants. This was surprising considering many positive changes to the aesthetic plastic surgery fellowship curriculum. Given that an additional 3 years have passed in this report. We have expanded on this analysis of aesthetic plastic surgery match statistics through the 2025 match cycle to see whether this trend held true. We again compared trends with competing facial plastic surgery and oculofacial surgery fellowships. In the present study, the authors show that aesthetic plastic surgery fellowship interest did not follow the previous trend. Instead applications dramatically increased, more than doubling from between 2022 and 2025.

In a recent study, Katz et al came to somewhat different conclusions when they compared National Resident Matching Program, ACGME, and SF Match data on applicant participation and match rates for otolaryngology, ophthalmology, dermatology, and plastic surgery residents pursuing aesthetic fellowships from 2013 to 2022.^13^ The authors identified cross-sectional percentages of graduating residents applying to, and matching into, aesthetic fellowships relative to other nonaesthetic fellowship options for their specialty for each given year. They concluded that the percentage of graduating residents pursuing aesthetic fellowships has significantly increased across time for all 4 residency specialties. They further reported that the highest percentage of aesthetic fellowship applicants graduated from “surgery-heavy” otolaryngology and plastic surgery residency programs and that a significantly larger proportion of otolaryngology graduates pursued facial plastic surgery fellowships than plastic surgery and other residency graduates. Important differences should be noted when our data is compared with the Katz et al study. Katz et al covered an earlier time period of 2013 to 2022, a majority of which predated the Aesthetic Plastic Surgery SF Match. Our study was designed to analyze the effect of the SF Match, and therefore, focused on the time period of 2018 to 2025. Unlike the analysis performed by Katz et al, we evaluated desirability of aesthetic subspecialization through the lens of fellowship position supply vs fellowship position demand. We did this by expanding SF Match data analysis to include absolute number of programs, available positions, participating applicants, match rates, and position fill rates across time for plastic surgery, otolaryngology, and ophthalmology. This allowed for more detailed statistical analyses that could appropriately compare aesthetic fellowship match trends between competing specialties. In addition, conclusions by Katz et al are limited by methodological issues, including the use of *t*-tests and ANOVA for proportional data that is not normally distributed, omission of logistic regression for count data, lack of temporal correlation, and absence of multiple testing corrections.

It remains unclear why there was a dramatic increase in aesthetic plastic surgery applications in the later part of our study period, and whether this trend will continue. It is likely that the efforts by The Aesthetic Society have had at least some positive effect. For example, additional changes mirroring residency efforts by the Plastic Surgery Residency Review Committee of the ACGME were approved and initiated in 2023. This included a standardized curriculum, standardized operative log, guaranteed standardized salary, vacation time, academic meeting time, and annual anonymous fellow and program director survey. In addition, The Aesthetic Training Committee established 2 working groups: The Fellowship Work Group to oversees individual program quality, and The Education Work Group to formalizing didactic teaching component of the fellowship. Anecdotally, discussions with prospective applicants suggest that many were unaware of the changes enacted by the Fellowship Training Committee. Time may have been needed for these improvements to be well recognized and for fellowship interest to increase. Katz et al hypothesize that increased popularity of aesthetic fellowship subspecialization may be influenced by cultural shifts such as social media, the impact of COVID-19 on altered body image perceptions, and a significant rise in aesthetic procedures and overall aesthetic medicine market growth.^13^ Perhaps that is the case. These factors may prove even more popular after 2025.

Postresidency aesthetic training is not unique to plastic surgery. It is also available to a number of other specialties. We have therefore also attempted to compare postgraduate plastic surgery aesthetic training to opportunities in facial plastic and oculofacial surgery including program numbers, fellowship, positions, and fellowship fill rates. In 2018, at the beginning of this study period, the total number of available positions in facial plastic and oculofacial surgery exceeded that of aesthetic plastic surgery by factors of 2.8 and 1.6, respectively. By 2022, the number of fellowship positions in facial plastic surgery exceeded that of aesthetic plastic surgery by a factor of only 1.3, whereas the number of positions in aesthetic plastic surgery actually exceeded that of oculofacial surgery by a factor of 1.6. The Aesthetic Society therefore appears to have succeeded in improving aesthetic plastic surgery fellowship opportunities for graduating plastic surgery residents in just 4 application cycles, with total aesthetic fellowship positions now significantly closer to facial plastic surgery and oculofacial surgery than they were before participation in the SF Match. Interestingly, facial plastic surgery fellowship and oculofacial surgery applications trends followed a different pattern to what was seen with aesthetic plastic surgery in this study. All comparisons of programs, positions, participating applicants, fill rates, and match rates remained remarkably consistent and competitive for both subspecialties throughout the study period. In fact, there were no statistically significant changes in any of these comparisons between 2018 and 2022 or between 2022 and 2024 for either facial plastic surgery or oculofacial surgery fellowships. Oculofacial surgery had the highest rate of applications to available positions and was the only one of the 3 programs that filled 100% of their positions on several occasions. This suggests a highly sought after and competitive program. It should be noted, however, that oculofacial surgery and facial plastic surgery have been established on SF Match since the 2014 and 2015 match cycles, respectively.

Barriers affecting graduating residents' decision to seek postgraduate aesthetic training still remain. A 2019 Association of American Medical Colleges survey documented that 43.9% of incoming medical students took 1 or 2 gap years before starting medical school, and 13.4% took 3 or 4.^14^ Further, plastic surgery integrated residency has become so competitive that an independent research year before applying for residency is commonplace.^15^ A number of studies have documented that integrated residents are more likely to seek fellowship training than independent graduates.^16-20^ Increased years of training, increased age, having 1 or more children, and outstanding student loans have all been shown to deter residents from pursing fellowship training.^16-20^ Clearly, training fatigue negatively effects the likelihood of choosing further fellowship training. By comparison, the standard length of training in otolaryngology and ophthalmology is 5 years. This shorter training period may make fellowship training more desirable. Furthermore, graduating plastic surgery residents have already received diversified and in-depth training in a wide variety of aesthetic procedures and may very well graduate residency fully prepared to enter aesthetic practice. The Plastic Surgery Residency Review Committee mandates that every resident participates in a wide variety of aesthetic operations. Minimum numbers for each of these “index cases” are strictly established. Unlike plastic surgery, there is no specific minimum operative requirement for otolaryngology residents beyond rhinoplasty. Ophthalmology residents similarly have a limited exposure.^1,13,21^ Therefore, fellowship training is perhaps more critical to most ophthalmology and otolaryngology graduates if cosmetic surgery is to comprise a significant portion of their practice. Further, although aesthetic plastic fellowships are entirely focused on cosmetic surgery and medicine, both facial plastic and oculofacial fellowships have significant reconstructive components in their curricula. In 2022, for example, only 40% of facial plastic surgery fellowships offered cosmetic surgery for a majority of total procedures, 50% reported even split between cosmetic surgery and reconstructive surgery, and 10% offered cosmetic surgery for small minority of total procedures.^13^ The portion of fellowship devoted to aesthetic surgery is quite variable as a result.^22^

Other factors may have influenced residents' decisions to pursue a given aesthetic surgery fellowship. Although fellowships have increased dramatically in recent years, the majority of these programs focus on breast and body. Many graduating residents develop significant expertise in breast and body aesthetic surgery during residency training. On the other hand, previous surveys have documented that a major reason graduating residents seek aesthetic fellowships is to gain further experience and expertise in facial aesthetics. Several means of addressing this dichotomy come to mind. The Aesthetic Training Committee might encourage additional fellowships focusing on facial aesthetic surgery. Another option might be to encourage current fellowships heavy in breast and body to arrange a formal several-month exchange with programs heavy in facial aesthetic training. This would possibly improve the fellowship experience for both programs involved.

Finally, variables such as economic, geographic (eg, city population, weather, proximity to applicant's home, desired long-term practice location), and other personal considerations may have affected these trends. Spending an additional year in training delays beginning formal plastic surgery practice, and therefore potential personal income. Although it may be assumed that fellowship training ultimately leads to long-term financial benefits, there is no data to support this. Significant salary deferment incurred during a fellowship year might be an unacceptable burden for graduating residents with existing educational loans, medical debt, childcare responsibilities, or other financial responsibilities. The COVID-19 pandemic occurred during the time period of our study and its effect on aesthetic postgraduate training is unclear. Although it is possible that the pandemic served to encourage fellowship training thereby delaying entry into practice, it may also have discouraged fellowship hiring. This remains an unstudied and unanswered question.

Graduating residents may also consider a fellowship alternative which provides both further aesthetic experience as well as an increase in personal income. By joining a group practices a junior member with senior associates, the graduating plastic surgery resident may believe they have the benefit of both worlds: an informal further educational experience as well as an increase in income.

Plastic surgeons are generally recognized as innovators in aesthetic surgery in large part because of support from our national societies, strong society, and member marketing efforts, an extensive series of national meetings, and top tier journals partially or exclusively dedicated to aesthetic medicine and surgery. In an effort to continue this legacy for the foreseeable future, we might look to strategies to maintain and expand our leadership profile. Of course, competition in aesthetic medicine and surgery is influenced by more than just fellowship training. Perhaps the best means of assuring that plastic surgery produces the best aesthetic product is to maintain a willingness to change and embrace process improvement at all levels, including the resident, postgraduate, and practicing plastic surgeon stages. This may mean exposure to aesthetic surgery earlier in residency training. It might also include the addition of more advanced aesthetic training to the later years of residency. Competency-based plastic surgery resident training is an ongoing ACGME-approved pilot program in plastic surgery that offers the opportunity for residents to complete plastic surgery residency training in 5 rather than 6 years.^17,23,24^ These residents, therefore, have the option of pursuing a postgraduate fellowship during what would have been their sixth year of residency training. Another means to provide additional opportunity for advanced aesthetic training is through focused training programs undertaken during the final year of residency. A proposal originating from the recently formed Aesthetic Committee of the ABPS suggested a pilot of “focused training in plastic surgery.”^25^ Given that senior residents generally have a clear idea of the direction of their future practice, this pilot is designed to give these residents the option of spending 4 to 6 months of their sixth year focused in the subspecialty area of their greatest interest.

Aesthetic plastic surgery training continues to evolve in a positive direction, both within plastic surgery residency and in postgraduate opportunities. With financial, educational, and career-specific considerations at play, fellowship programs have continued to adapt in order to meet the evolving needs and preferences of prospective fellows. Some of the competing factors which we have outlined may lead to training fatigue and mitigate against further training, underscoring the nuanced nature of posttraining decision making. Variability in fellowship experience and applicant goals makes it difficult to define a singular “perfect” aesthetic plastic surgery fellowship program. That being said, The Aesthetic Committee has appropriately outlined characteristics that programs must offer to provide a universally positive fellowship experience, including a competency-based curriculum that integrated advanced aesthetic surgical and nonsurgical techniques of the face, breast, and body, personalized mentorship from leading surgeons, fair financial support, and a fellow clinic that encourages graduated autonomy. Strategic interventions are actively being implemented to enhance the appeal of aesthetic plastic surgery fellowship programs and to continue attracting more applicants. Flexible application processes, virtual or hybrid interviews, and consideration of geographic preferences can further improve accessibility for prospective applicants. Programs should increase visibility and relevance through collaboration with The Aesthetic Society, leveraging national meetings, journals, and digital platforms to showcase alumni success and unique training opportunities. Addressing training fatigue may require innovative models to demonstrate the value of advanced aesthetic fellowship training. Financial burdens can be mitigated through desirable salaries and stipends and loan repayment options. Enhancing curricula, interdisciplinary exposure, and advanced simulation tools may further differentiate aesthetic surgery fellowships from residency training. Finally, programs should conducting, evaluating, and responding to resident surveys to better understand motivations for pursuing aesthetic surgery fellowship, as well as graduating fellow survey to reflect on successes and areas for improvement.

### Limitations

The decision to apply to a fellowship is complex and multifaceted. We can speculate on the factors that might drive this decision. However, each individual will have their own set of reasons. Although this analysis provides valuable insights, the lack of data on resident attitudes limits the scope of conclusions, warranting caution in generalizing findings. For example, a lack of enthusiasm for some residents to pursue aesthetic training may be because of recognized or unrecognized biases developed during academic residency training. A number of variables that could not be analyzed with SF Match data may have affected these trends, including economic conditions, geographic preferences, the COVID-19 pandemic, family dynamics, and graduating residents' personal needs. We have therefore hypothesized on how these variables might have affected graduating residents' choices to seek or not to seek fellowship training. This study lacks survey data from prospective applicants, active fellows, and alumni, which would be valuable to better understand these trends. Future studies will survey attitudes and motivations of plastic surgery residents regarding fellowship training to better understand the drivers of their decision making and identify actionable solutions. This study does not explicitly explore the attitudes of residents toward fellowship applications, but we hope to survey senior residents and active fellows on this specific topic in the near future. Perhaps understanding these influences will help guide fellowship program design at large.

We were unable to evaluate nonendorsed aesthetic plastic surgery fellowships because there is no data repository for fellowships that do not participate in the SF Match. As a result, we may be underestimating the number of aesthetic plastic surgery fellowship programs and trainees. Similarly, we cannot distinguish between programs created after 2018 and preexisting aesthetic plastics surgery fellowships which joined the SF Match during the course of the study. The SF match and other fellowship changes noted were initiated in 2018 limiting our analysis to the years since these changes were made. Because this represents a relatively short time period, there is no guarantee that the trends noted will continue as described. It should be pointed out that it would have been helpful to evaluate ratios of participating applicants to available fellowship positions over time, but these statistical analyses were not performed. This may have helped to depict changes in competitiveness over time and demonstrated disproportionate changes in applicants relative to available spots between specialties. Finally, it would have been very helpful to analyze breakdown of each applicant's rank list to further elucidate preference trends and general program reputations, although this data was not available. Despite these limitations, we feel the data presented provide a meaningful and actionable update on the state of fellowship training in aesthetic surgery.

## CONCLUSIONS

The number of postgraduate aesthetic fellowship programs and positions increased dramatically during the early part of the 2018 to 2025 study period. However, it was only in the later part of this study that a significant increase in aesthetic plastic surgery applicants was observed. It remains unclear whether this increase in interest will be sustained in the future or merely represents a temporary variant. The discrepancy in program and applicant numbers narrowed when postgraduate aesthetic surgery fellowships were compared with facial plastic surgery. Oculofacial surgery fellowship program numbers were essentially unchanged over the study period, but consistently remained in high demand as demonstrated by the high fill rate. Definitive reasons for the increase in aesthetic plastic surgery applications will require direct insight from current and prospective fellows.

## Supplemental Material

This article contains supplemental material located online at https://doi.org/10.1093/asjof/ojaf123.

## Supplementary Material

ojaf123_Supplementary_Data
