# Supplementary material for: Comparing Temporal Trends in Aesthetic Surgery Fellowship Match Statistics in Plastic Surgery, Facial Plastic Surgery, and Oculofacial Surgery
Source: Aesthet Surg J Open Forum. 2025 Oct 4;7:ojaf123. doi: 10.1093/asjof/ojaf123 (PMC12614165; doi:10.1093/asjof/ojaf123)
Supplement: ojaf123_Supplementary_Data [file ojaf123_supplementary_data.zip › Supplemental Table 1 - PRS Graduates.docx]

| Year | Graduating Residents | IRR | 95% CI | p-value |
| --- | --- | --- | --- | --- |
| 2018 | 200 | - | - | 0.71 |
| 2019 | 208 | 1.04 | 0.86, 1.26 |  |
| 2020 | 213 | 1.07 | 0.88, 1.29 |  |
| 2021 | 221 | 1.11 | 0.91, 1.34 |  |
| 2022 | 225 | 1.13 | 0.93, 1.36 |  |
| 2023 | 230 | 1.15 | 0.95, 1.39 |  |

**Supplemental Table 1.** Trends in the Number of Plastic Surgery Residents Over Time, 2018 – 2023.
