# Supplementary material for: Comparing Temporal Trends in Aesthetic Surgery Fellowship Match Statistics in Plastic Surgery, Facial Plastic Surgery, and Oculofacial Surgery
Source: Aesthet Surg J Open Forum. 2025 Oct 4;7:ojaf123. doi: 10.1093/asjof/ojaf123 (PMC12614165; doi:10.1093/asjof/ojaf123)
Supplement: ojaf123_Supplementary_Data [file ojaf123_supplementary_data.zip › Supplemental Table 2 - ENT Graduates.docx]

| Year | Graduating Residents | IRR | 95% CI | p-value |
| --- | --- | --- | --- | --- |
| 2018 | 311 | - | - | 0.86 |
| 2019 | 315 | 1.01 | 0.87, 1.18 |  |
| 2020 | 331 | 1.06 | 0.91, 1.24 |  |
| 2021 | 327 | 1.05 | 0.90, 1.23 |  |
| 2022 | 334 | 1.07 | 0.92, 1.25 |  |
| 2023 | 340 | 1.09 | 0.94, 1.28 |  |

**Supplemental Table 2**. Trends in the Number of Graduating Otolaryngology Residents Over Time, 2018 – 2023.
