# Supplementary material for: Comparing Temporal Trends in Aesthetic Surgery Fellowship Match Statistics in Plastic Surgery, Facial Plastic Surgery, and Oculofacial Surgery
Source: Aesthet Surg J Open Forum. 2025 Oct 4;7:ojaf123. doi: 10.1093/asjof/ojaf123 (PMC12614165; doi:10.1093/asjof/ojaf123)
Supplement: ojaf123_Supplementary_Data [file ojaf123_supplementary_data.zip › Supplemental Table 3 - Ophtho Graduates.docx]

| Year | Graduating Residents | IRR | 95% CI | p-value |
| --- | --- | --- | --- | --- |
| 2018 | 499 | - | - | 0.94 |
| 2019 | 477 | 0.96 | 0.84, 1.08 |  |
| 2020 | 501 | 1.00 | 0.89, 1.14 |  |
| 2021 | 502 | 1.01 | 0.89, 1.14 |  |
| 2022 | 509 | 509 | 0.90, 1.15 |  |
| 2023 | 501 | 1.00 | 0.89, 1.14 |  |

**Supplemental Table 3.** Trends in the Number of Graduating Ophthalmology Residents Over Time, 2018 – 2023.
